# Lumps and Bumps on the Abdominal Wall: Role of Fine Needle Aspiration Cytology

**DOI:** 10.5146/tjpath.2023.12926

**Published:** 2024-05-18

**Authors:** Anjali Ashtakar, Asha Shenoy, Mona Agnihotri, Kanchan Kothari

**Affiliations:** Department of Pathology, Seth G.S. Medical College and King Edward Memorial Hospital, Mumbai, India

**Keywords:** FNAC, Abdominal wall, Neoplasm, Lipoma, Metastasis

## Abstract

***Objective: *** Abdominal wall masses often pose diagnostic challenges for clinicians due to their nonspecific symptoms. They include a wide spectrum of lesions ranging from inflammatory to tumor-like masses and malignancies. The majority of the malignant nodules are metastatic in origin and may be the initial presentation of a primary malignancy; hence, an early diagnosis is important. Fine-needle aspiration cytology (FNAC) is a valuable diagnostic tool in the evaluation of such lesions.

This was a retrospective study of the cytomorphological spectrum of abdominal wall masses, conducted at a tertiary health care centre over a three-year period.

***Material and Methods:*** The study included patients of all age groups presenting with an abdominal wall mass. These lesions were assessed by FNAC. The diagnosis was made on cytology smears and subsequently correlated with the histopathological diagnosis, wherever possible.

***Results: *** Of the 70 cases, 21 were non-neoplastic and 49 neoplastic. A benign neoplasm was the most common lesion (52.9%), followed by non-neoplastic lesions (30%) and malignant neoplasms (17.1%). Lipoma was the most common benign neoplasm and metastasis was the commonest malignant neoplasm. The most common type of tumor metastasising was adenocarcinoma and the primary tumors were predominantly intra-abdominal. No false-negative results were seen.

***Conclusion:*** Most of the abdominal wall masses display a characteristic cytomorphology, which needs to be identified and recognized by a cytopathologist for an accurate diagnosis. FNAC plays an invaluable role in the detection of metastases, especially at sites such as the umbilicus, which may be the only manifestation of an underlying advanced malignant disease.

## INTRODUCTION

Abdominal wall masses often pose diagnostic challenges for clinicians due to their nonspecific symptoms. They include a wide spectrum of lesions ranging from inflammatory to tumor-like masses and malignancies (1). The majority of the malignant nodules are metastatic in origin and may be the initial presentation or a sign of widespread disease of a primary malignancy; hence, an early diagnosis is important (2). Fine Needle Aspiration Cytology (FNAC) is a simple, fast and non-invasive diagnostic modality which can be performed with ease on abdominal wall lesions for appropriate patient management (3). Most of the abdominal wall masses display a characteristic cytomorphology, which needs to be identified and recognized by a cytopathologist for an accurate diagnosis. A literature search showed sparse publications on the comprehensive study of cytologic features of abdominal wall masses. Hence, this study was undertaken to find out the spectrum of abdominal wall masses and the efficacy of FNAC in the early diagnosis of such lesions.

## MATERIALS and METHOD

The present study was conducted on 70 patients with abdominal wall masses at a tertiary health care centre over a 3-year period. FNAC was done by the standard technique using a 22 to 23 G needle attached to a 5 ml disposable syringe. Air-dried and wet fixed smears were prepared and stained with Giemsa and Papanicolaou stains, respectively. Special stains and immunocytochemistry (ICC) were done wherever required. Cytomorphological features were analyzed and correlated with clinical and radiological findings, and histopathology, wherever available.

## RESULTS

In the present study, the age ranged from 16 to 70 years with maximum number of cases seen the 21-30 years followed by the 31-40 years age group and the male to female ratio was 1:1. Of the 70 cases, 65 cases were aspirated from the abdominal wall, with the right lumbar region being the most frequent site of aspiration, and 5 cases from the umbilical region.

The diagnoses were classified as non-neoplastic lesions including inflammatory lesions with 21 (30%) cases and neoplastic lesions with 49 (70%) cases, respectively. Out of the neoplastic lesions, 37 (52.9%) were benign and 12 (17.1%) were malignant. Thus, in our study, the most common category of the lesion was benign neoplasms (52.8%), followed by non-neoplastic lesions (30%), and malignant neoplasms (17.1%).

**Figure 1:**
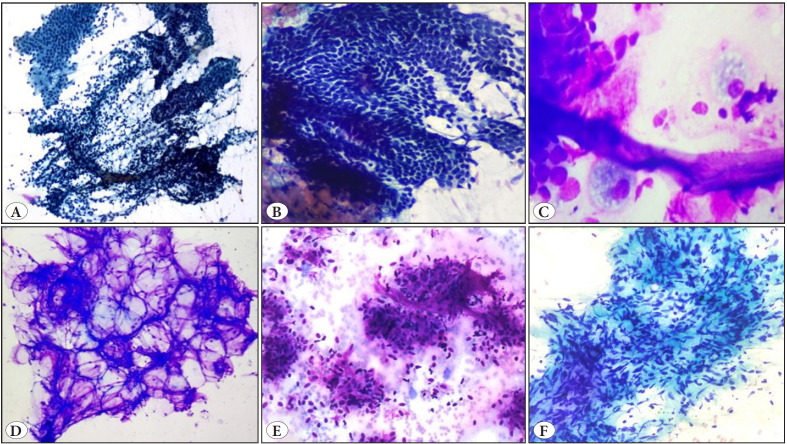
**A-C)** Endometriosis (Pap stain, x100, x400; Giemsa stain, x400) **D)** Lipoma (Giemsa stain, x100) **E)** Benign Spindle cell lesion of fibroblastic/myofibroblastic origin. (Giemsa stain, x400). **F)** Benign spindle cell lesion of neurogenic origin (Pap stain, x400).

Out of the 21 non-neoplastic cases, 12 cases were inflammatory lesions, seven endometriosis, and two were keratinous cysts. Inflammatory lesions consisted of acute inflammation, necrotizing inflammation, granulomatous inflammation, foreign body giant cell reaction, and cysticercosis. Abdominal wall endometriosis/scar endometriosis was seen in patients with a mean age of 32 years and all presented with a palpable abdominal nodule and pain. Cyclic pain was present in five cases and there was a history of prior caesarean section (abdominal scar) in six cases. Smears showed varying cellularity comprising epithelial cells (7cases), spindle stromal cells (4 cases), and hemosiderin-laden macrophages (2 cases) (Figure 1). A cytologic diagnosis of endometriosis was rendered in all seven cases and histopathologic confirmation was available in one case while the rest of the cases responded to medical treatment.

Of the 37 benign neoplasms, 29 cases were of lipoma and this was the most common benign lesion encountered in our study. Eight cases of benign spindle cell lesion included 5 cases of undetermined type, 2 cases of myofibroblastic/fibroblastic origin suggestive of desmoid tumor, and 1 case of neurogenic origin (Figure 1). Histopathology was available in three cases of undetermined spindle cell lesion, which turned out to be desmoid tumor, schwannoma, and fibrolipoma.

**Figure 2:**
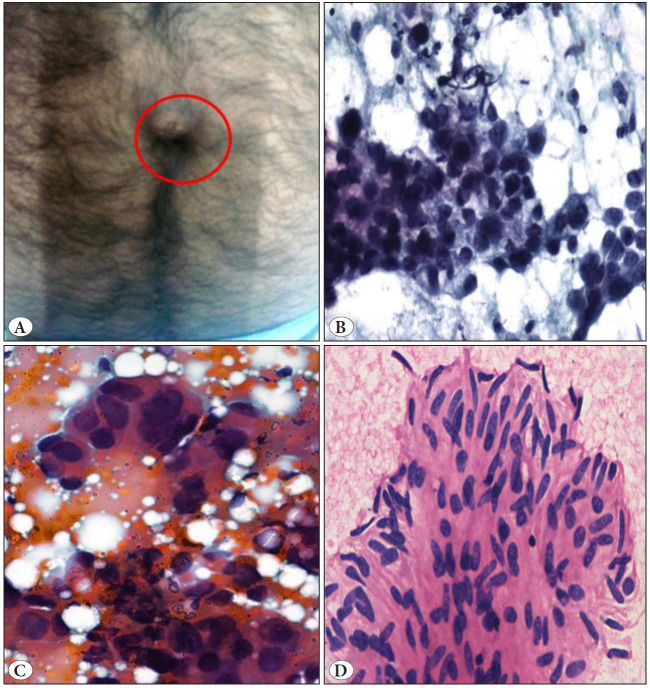
Sister Mary Joseph’s Nodule: **A)** Clinical picture - Umbilical nodule. **B,C)** Pleomorphic cells in clusters and ill-defined glands. (Giemsa, x400). **D-F)** Metastasis of Gastrointestinal stromal tumor (H&E, x100, x400).

Out of the 12 malignant cases, 11 were metastatic lesions and a single case was of primary tumor (pleomorphic sarcoma). Metastatic deposits included six cases from adenocarcinoma, two from renal cell carcinoma (RCC), and one case each from melanoma, infiltrating duct carcinoma (IDC), and malignant gastrointestinal stromal tumor (GIST). A known primary in gallbladder was present in two cases of metastatic adenocarcinoma. Thus, metastasis from the known primary was seen in seven cases, of which five cases were intra- abdominal (gallbladder-2, RCC-2, malignant GIST of stomach-1). In the present study, four nodules, universally called Sister Mary Joseph’s nodule (SMJN), were located at the umbilicus, out of 11 metastatic cases (Figure 2). Cytomorphological features from umbilical metastasis showed adenocarcinoma in three cases and atypical spindle cells in one case. The primary site was gallbladder in two cases (Figure 2) and malignant GIST in one case (Figure 2). The third case, of adenocarcinoma, underwent a biopsy of the abdominal wall nodule. The biopsy was consistent with the cytologic diagnosis. Immunohistochemistry (IHC) showed positivity for CK7 and was negative for CK20, TTF-1, PAX-8, and CDX-2. The possible site of primary suggested was the upper gastrointestinal tract (GIT)/pancreaticobiliary tree. Thus, SMJN was a presenting symptom in one case with internal malignancy and represented a recurrence in three cases.

**Figure 3:**
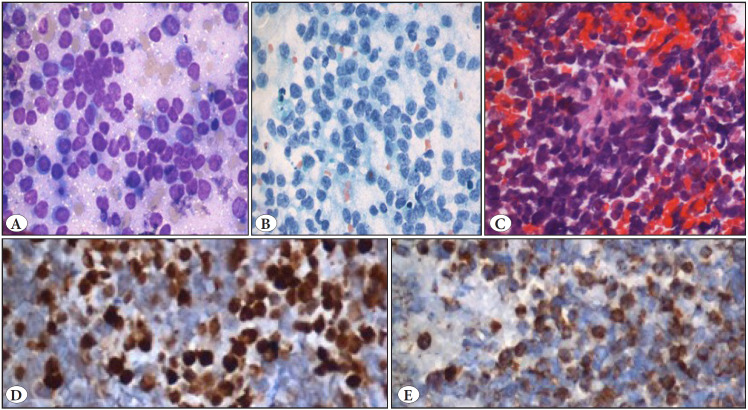
Metastatic Melanoma (Amelanotic): **A,B)** Malignant round cells with high N: C ratio and prominent nucleoli. (Giemsa stain, x400; Pap stain, x400). **C)** Cell block showing malignant cells (HE, x100). **D,E)** Immunohistochemistry: S100 and HMB 45 positive.

Our study had a case of metastasis of amelanotic melanoma, and FNAC smears showed dispersed malignant cells with round cell morphology. Poorly differentiated carcinoma, lymphoma, and rhabdomyosarcoma were included as differential diagnoses. Melanoma was eliminated from the differential due to the absence of pigment and prominent nucleoli. On inquiry, the patient gave a history of previous surgery for nasal melanoma. Cell block with IHC showed positivity for S100 and HMB 45, thus confirming the primary site (Figure 3). In our study amongst two metastatic RCC, one of the patients had a nodule at the nephrectomy scar site. FNAC showed clusters of tumor cells with abundant eosinophilic granular to vacuolated cytoplasm and adherent delicate fibrovascular cores and cells were positive for CD 10 on immunocytochemistry (ICC).

Cytopathological and histopathological correlation was available in ten cases (14.3%). Out of these ten cases, five cases were from lipoma and four of benign spindle cell lesions, and one was non-neoplastic (endometriosis). All Inflammatory lesions responded to medical treatment. Of the 11 cases of metastases, the primary site was known in eight cases and thus biopsy was not done. The rate of histopathology follow up was low in our study as lipoma was the most common lesion and it is usually not excised.

## DISCUSSION

Abdominal wall masses and mass-like lesions are a common clinical presentation and often incidental findings at cross-sectional imaging. Detailed clinical history and imaging findings can be triaged with FNAC for a definitive diagnosis. A variety of non-neoplastic and neoplastic lesions can be encountered in the abdominal wall region. Non-neoplastic lesions include suture granuloma, abscess, keloid, endometriosis, hematoma, and epidermoid cyst. Neoplasms may be primary (benign or malignant) or metastatic (1).

The most common category of the abdominal wall masses is benign neoplasms followed by non-neoplastic lesions and malignant neoplasms, with lipoma being the commonest, as seen in the present study (4). However, Rana et al. have found metastasis as the most common lesion, followed by a lipoma and cysticercosis (3). In some studies, the desmoid tumor has been described as the most common soft tissue tumor in the abdominal wall but this may be because lipomas are not usually treated surgically and were excluded from the analysis.

Abdominal wall endometriosis (AWE) usually develops in a surgical abdominal scar after gynecological surgeries like caesarean section, as seen in most of our cases. An increase in the size of the lump, bleeding and skin discoloration with cyclical changes of menstruation are pathognomonic of scar endometriosis (5). The typical symptoms are often absent and this can be a diagnostic pitfall for clinicians. It can be mistaken for a hernia, suture granuloma, desmoid tumor, nodular fasciitis, lipoma, sarcoma, or metastatic malignancy. FNAC is a promising tool for rapid and accurate pre-operative diagnosis (6). It shows characteristic cytological features like epithelial cells, spindle stromal cells, and a variable number of hemosiderin-laden macrophages with inflammatory cells. The presence of any two of the three components has been used for the cytological diagnosis of endometriosis (7).

Primary malignant cutaneous and subcutaneous tumors of the abdominal wall are very rare (2). The majority of malignant nodular lesions in the abdominal wall are metastatic tumors, which usually indicates advanced tumor stage or may sometimes be the initial presentation of an underlying malignancy (3). The overall incidence of metastasis in our study was 15.71%, lower than Rana et al. (36.9%) but higher than Kumar et al. (0.5%) and Dash et al. (0.7%) (3,4,8). The reason for this low incidence could be that the enlisted studies dealt with the FNAC of all palpable subcutaneous nodules rather than the specific site as done in our study. The literature describes the abdominal wall as the single most frequent site for appearance of metastases from an unknown or a known primary (9). The most common type of tumor metastasising is adenocarcinoma (10). Metastasis usually tends to occur close to the site of the primary malignancy i.e., most of the abdominal wall/ umbilical metastases were from intra-abdominal, pelvic organs or retroperitoneum (3). This was confirmed by our study, where six out of eight metastatic lesions of known primary were intra-abdominal and the most common primary was the gallbladder followed by RCC. David et al. also had a similar observation (2).

Umbilical metastasis from intra-abdominal carcinomas, also called Sister Mary Joseph’s nodule (SMJN), is well documented. It may be the only manifestation of an underlying advanced malignant disease and therefore clinicians should be aware of such nodules. The umbilical nodule may be the presenting symptom in patients with internal malignancies or it may represent a late finding in patients with a disseminated disease (11). In our study also, SMJN was a presenting symptom in one case with internal malignancy and represented a recurrence in three cases and all were intra-abdominal malignancies. The reason for the umbilicus to be an easy target for metastasis from an intra-abdominal tumor is because of its embryological development. The spread may either be contiguous, from intraperitoneal metastasis via the portal vein, or by retrograde lymphatic flow from the inguinal lymph nodes (12,13).

Scar-site metastasis is very rare, accounting for 0.9-1.8% following conventional open nephrectomies, and implies a poor prognosis (14). The present study had a case of scar-site metastasis of RCC which was diagnosed on cytology. FNA, indeed, is the investigation of choice for the detection of a suspicious lesion at superficial sites.

## CONCLUSION

FNAC is the first line of investigation to evaluate abdominal wall masses and can help in the definitive diagnosis, thus avoiding unnecessary surgical intervention in the majority of cases. It is a rapid technique for the detection of metastatic lesions at the superficial subcutaneous sites and at the umbilicus which may be the only manifestation of an underlying advanced malignant disease. Adequate FNAC sampling, preserved cytomorphology, and complete clinical details are prerequisites for avoiding false-negative results.

## Conflict of Interest

No conflict of interest in connection to the article submitted.

## Funding

No funding.

## Ethics Approval

The study was approved by the Institutional ethics committee (IEC) with a waiver of informed consent (IEC No.EC/54/2019).
